# BananaImageBD: A comprehensive banana image dataset for classification of banana varieties and detection of ripeness stages in Bangladesh

**DOI:** 10.1016/j.dib.2024.111239

**Published:** 2024-12-19

**Authors:** Md Hasanul Ferdaus, Rizvee Hassan Prito, Ahmed Abdal Shafi Rasel, Masud Ahmed, Md. Jahid Hassan Saykot, Shanjida Sultan Shanta, Sonali Akter, Ankan Chandra Das, Mohammad Manzurul Islam, Mahamudul Hasan, Md Sawkat Ali

**Affiliations:** a Department of Computer Science and Engineering, East West University, Dhaka, Bangladesh; b Department of Agricultural Extension, Ministry of Agriculture, Bogura, Bangladesh

**Keywords:** Machine learning, Computer vision, Image classification, Object detection, Horticulture, Food processing, Precision agriculture, Deep learning

## Abstract

Bananas are among the most widely consumed fruits globally due to their appealing flavor, high nutritional value, and ease of digestion. In Bangladesh, bananas hold significant agricultural importance, being one of the most extensively cultivated fruits in terms of land coverage and ranking third in production volume. The banana image dataset presented in this article includes clear and detailed images of four common banana varieties in Bangladesh: Sagor Kola (*Musa acuminate*), Shabri Kola (*Musa sapientum*), Bangla Kola (*Musa* sp.), and Champa Kola (*Musa sapientum*), as well as four key stages of banana ripeness: Green, Semi-ripe, Ripe, and Overripe. The bananas were collected from wholesale markets and retail fruit shops located in different places in Bangladesh. Overall, the dataset has 2471 original images of different varieties of bananas and 820 original images of varying ripeness stages of bananas. All the images were carefully captured using a high-quality smartphone camera. Later, each image was manually reviewed, maintaining the quality standard throughout the dataset. The augmented version of the banana variety classification dataset contains 7413 images and the augmented banana ripeness stages dataset contains 2457 images. The dataset possesses immense potential in driving innovation and development of automated and efficient processes and mechanisms in several fields, including precision agriculture, food processing, and supply chain management. Machine Learning (ML) and Deep Learning (DL) models can be trained on this dataset to accurately categorize banana varieties and determine their ripeness stages. Such ML and DL models can be leveraged to develop automated systems to determine the optimal harvest time, establish standards for quality control of bananas, develop products and marketing strategies through analysis of consumer preferences for various banana varieties and ripeness levels, and streamline the banana supply chain through improvements in harvesting, sorting, packaging, and inventory management. Additionally, researchers aiming to contribute to developing Computer Vision technologies in food and agricultural sciences will find this dataset valuable in advancing precision farming and food processing mechanisms. Therefore, the dataset has a vast capacity for automating banana production and processing, minimizing the costs of manual labor, and improving overall efficiency.

Specifications Table

**Table:** 

Subject	Computer Sciences, Agricultural Sciences, Biological Sciences
Specific subject area	Machine Learning and Computer Vision-based automated classification of banana varieties and detection of ripeness stages.
Type of data	RGB Images (raw and augmented) having 256×256 dimensions in JPEG format.
Data collection	Images of four sides of each banana were captured using smartphone cameras after purchasing the bananas from various local markets in Bangladesh. Before taking the photo, each banana was cut out from the stem and placed on a white page under a white LED light to prevent background-related distortions and color variations in images. Images were taken with smartphone cameras of high resolution. The data collection team categorized each image and the corresponding ripeness level of the banana object under expert supervision from the agricultural field. Each image was manually checked for quality-related issues, and poor-quality images were filtered out. In the next step, some augmentation techniques were applied to increase the dataset and ensure that variance in the real-world data can be dealt with effectively.
Data source location	The images were sourced from the following places in Bangladesh: • Karwan Bazar, Dhaka, Bangladesh (23.75228° N, 90.39438° E); • Meradia Kacha Bazar, Dhaka, Bangladesh (23.76287° N, 90.44472° E); • Dhaka New Market, Dhaka, Bangladesh (23.73327° N, 90.38395° E); • Rangpur City Bazar, Rangpur, Bangladesh (25.75371° N, 89.25349° E).
Data accessibility	Repository name: BananaImageBD: A Comprehensive Image Dataset of Common Banana Varieties with Different Ripeness Stages in Bangladesh. Data identification number: 10.17632/ptfscwtnyz.2 Direct URL to data: https://data.mendeley.com/datasets/ptfscwtnyz/2
Related research article	*None*

## 1 Value of the Data

• *Computer Vision and Image Processing***:** This dataset can be useful for training machine learning models for classifying various banana varieties and detecting their ripeness levels. Moreover, such models can be further trained to detect and locate individual bananas within images and videos, a feature crucial for developing robotic harvesting systems [1].
• *Precision Agriculture***:** This dataset serves as a valuable tool for accurately identifying various banana varieties and developing automated systems to determine the optimal harvest time by assessing their ripeness levels. This has the potential to significantly enhance the efficiency of processes ranging from cultivation to harvesting, and storing and selling [2], ultimately reducing labor costs and increasing overall productivity.
• *Food Science and Technology*: The dataset can be leveraged by food industries to establish standards for the purpose of quality control of bananas considering their color, texture, and appearance. Accurate identification of ripeness can reduce errors in harvesting and increase efficiency in food processing. Furthermore, product development and marketing strategies can be benefited through analysis of consumer preferences for various banana varieties and ripeness levels.
• *Supply Chain Optimization***:** Automated systems detecting ripeness stages can streamline the banana supply chain. This includes improvements in harvesting, sorting, packaging, and inventory management, ensuring that bananas reach the market at the ideal stage of ripeness. Such efficiencies help minimize waste and improve customer satisfaction [3].
• *Economic Benefits***:** Minimizing the risks of premature harvesting and spoilage from over ripeness can provide economic advantages. Farmers, distributors, and retailers all stand to benefit from reduced costs, improved efficiency, and higher profits; thereby, contributing to the broader growth of the agricultural industry and economy.
• *Research and Development Resource***:** The potential applications of this dataset extend beyond immediate agricultural use. It could support the development of tools and systems that assist in selecting bananas at preferred ripeness levels, whether through mobile applications or in-store technologies. Such advancements would enhance the consumer experience and contribute to reducing food waste.

## 2 Background

Bananas are herbaceous plants with a pseudostem, underground rhizomes, and broad and elongated leaves. They produce flowers in a pendulous inflorescence which develop into clusters of seedless, elongated fruits. Bananas thrive in warm climates that have well-drained soils and consistent moisture. They grow quickly and continuously produce new shoots, yielding abundantly under optimal conditions. The four most common varieties of bananas grown and consumed in Bangladesh are Sagor, Shabri, Bangla, and Champa. Other less common and rare varieties include Bichi, Kacha/Anaji, Agniswar, and BARI-1 [4]. Existing image datasets relating to bananas address aspects such as banana varieties [5], post-harvested banana tiers [6], leaves and stems for disease classification [[7], [8], [9], [10]]. To the best of our knowledge, there is no publicly available dataset for documenting banana ripeness stages. Our dataset addresses this gap by providing images of the four most common banana varieties in Bangladesh with the four key ripeness stages: Green, Semi-ripe, Ripe, and Overripe. This dataset has the potential to facilitate the automation processes of identifying banana varieties with their ripeness levels using computer vision with high accuracy in food processing. Optimal ripeness is essential for producing banana-based products. This dataset can enhance efficiency in banana industries, boost the economy, and support food security and sustainability globally. A detailed list of utility and potential usage of this dataset is presented in the VALUE OF THE DATA section.

## 3 Data Description

Our dataset provides two main sets of images: one for classifying common banana varieties and another for identifying the different stages of banana ripeness. We have also included augmented versions for both the datasets. The whole dataset is organized into four main folders (uploaded as ZIP compressed files on Mendeley Data [11]):

1. *Banana Classification Dataset***:** This folder contains images of different banana varieties. We have organized 2471 raw images into four subfolders, each named after a specific variety of banana in Bengali language: Bangla Kola, Champa Kola, Sabri Kola, and Sagor Kola. Each subfolder includes images of that banana variety at various stages of maturity.
2. *Augmented Banana Classification Dataset***:** This folder is an enhanced version of the first, containing 7413 images distributed across four subfolders named after the banana varieties (similar to Banana Classification Dataset). These images have been augmented to provide more varieties for training models, including flipped images, rotated images, and those with adjusted brightness, blur, or noise.
3. *Banana Ripeness Detection Dataset***:** This folder includes 820 images, categorized into four subfolders based on four ripeness stages: Green, Semi-ripe, Ripe, and Overripe. Each subfolder contains images of bananas at different stages of ripeness, labeled according to the specific stage they represent.
4. *Augmented Banana Ripeness Detection Dataset***:** Similar to the second folder, this folder contains an enhanced version of the ripeness dataset having 2457 images. It follows the same subfolder structure as the original ripeness dataset, with augmented versions of the original images.

All the images in our dataset are in JPEG format, with a resolution of 256×256 pixels and 96 dpi. Each image features a clear view of a banana against a white background. The augmented images include various modifications, such as flipping horizontally and vertically, rotating 90° clockwise, counterclockwise, and upside down, and more minor rotations within a range of −15° to +15°. We've also applied brightness, blur, and noise changes to add variety.

**Fig. 1:**
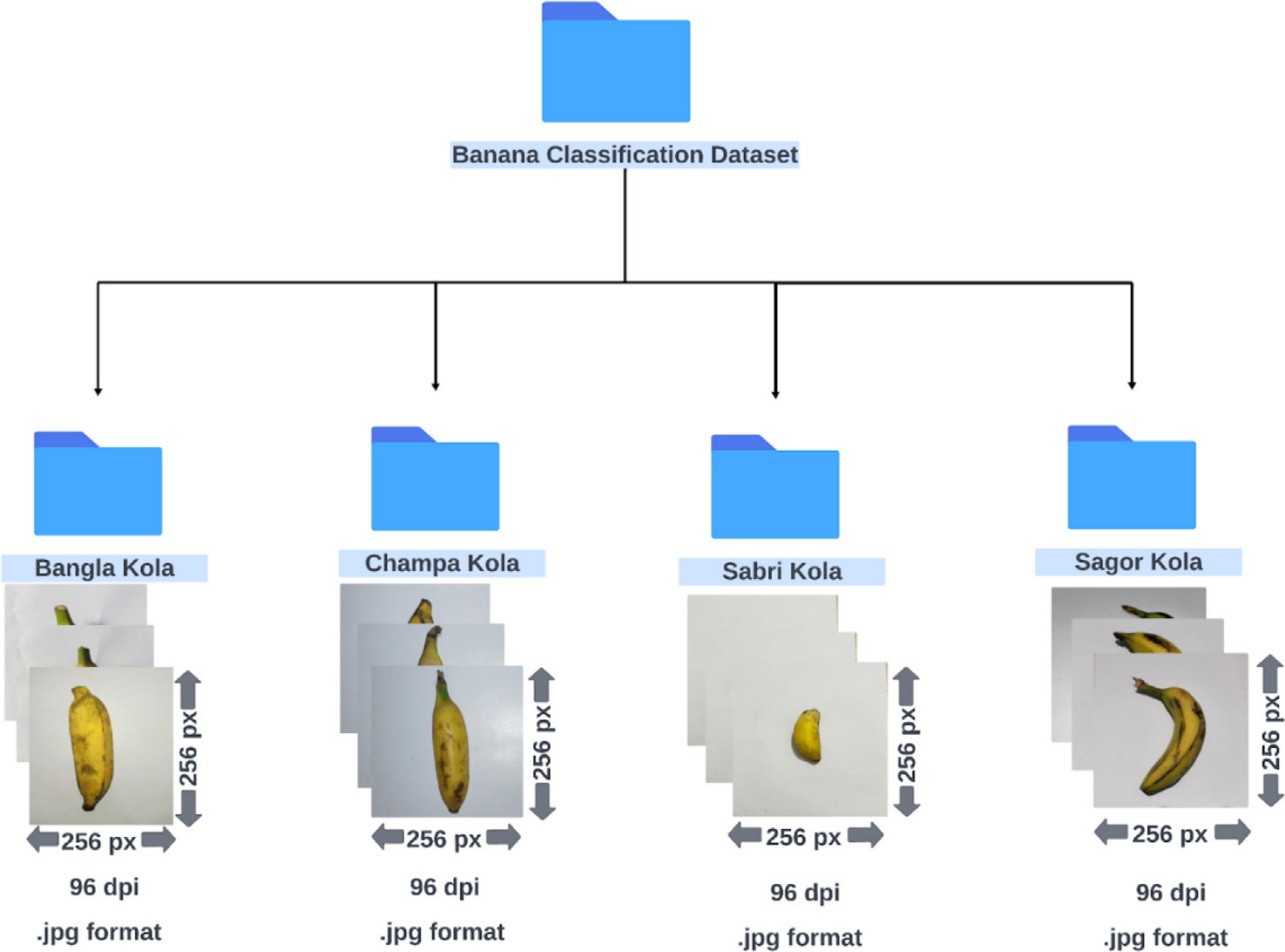
Folder structure of the Banana Classification Dataset.

**Fig. 2:**
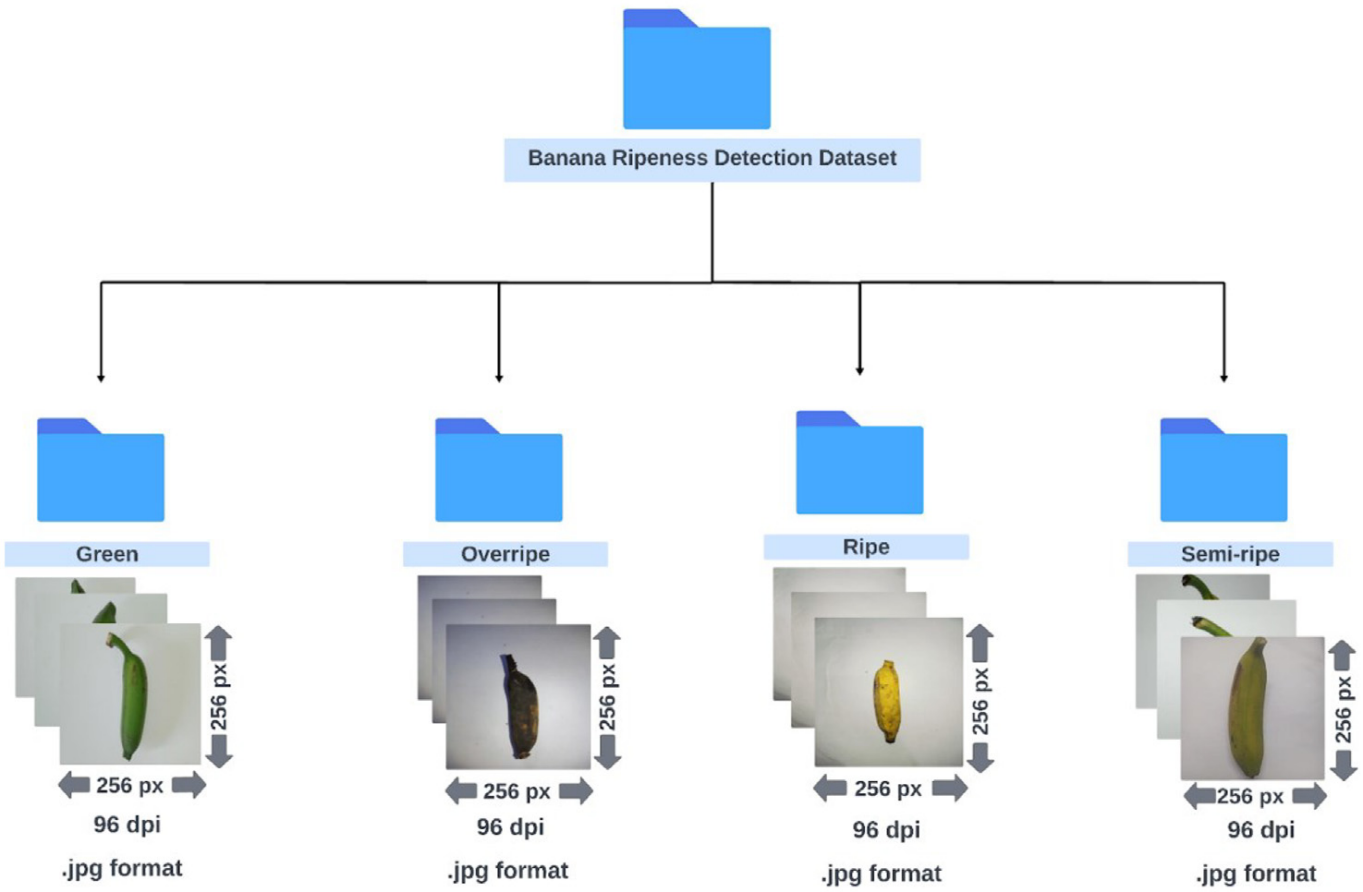
Folder structure of the Banana Ripeness Detection Dataset.

Figs. 1 and 2 show the folder structure of the original datasets and Table 1 provides further detailed information. We have compressed the files into a ZIP format, which helps save space and speeds up the process. Description and sample images of each variety of banana and each ripeness stage of banana included in our datasets are provided in Table 2.

**Table 1:** Detailed information of the datasets.

Dataset name	Class	Number of images	Total Size of the Dataset on Disk	Compressed Size of the Dataset
Banana Classification Dataset	Bangla Kola	444	17.2 MB	11.6 MB
	Champa Kola	994		
	Sabri Kola	509		
	Sagor Kola	524		
	**Total**	**2471**		
Augmented Banana Classification Dataset	Bangla Kola	1332	78.5 MB	63.7 MB
	Champa Kola	2982		
	Sabri Kola	1527		
	Sagor Kola	1572		
	**Total**	**7413**		
Banana Ripeness Detection Dataset	Green	212	5.55 MB	3.51 MB
	Semi-ripe	204		
	Ripe	201		
	Overripe	203		
	**Total**	**820**		
Augmented Banana Ripeness Detection Dataset	Green	636	25.2 MB	20.1 MB
	Semi-ripe	609		
	Ripe	603		
	Overripe	609		
	**Total**	**2457**

**Table 2:** Description and sample images of each category of the dataset.

Dataset	Class name	Description	Sample Images
Banana Classification Dataset	Sabri Kola	This type of banana is scientifically known as *Musa sapientum.* Alternative local names in Bangladesh are Malbhog, Onupam, and Martaman. This type of banana can be short to medium in size. They possess ivory-yellow color in the ripe stage and have a strong texture. Such bananas are covered with thin peels and manifest a sweet and tasty flavor [12].	
	Sagor Kola	Scientific name is *Musa acuminate.* Native names include Sagor Kola, Amrito Sagar Kola, Ranging Sagar Kola, Meher Sagar, and Agnisor. This variety of banana can be medium to large in size. Ripe bananas of this type expose bright yellow color and possess a pleasant taste [12].	
			
			
	Bangla Kola	Scientifically known by *Musa* sp. These bananas are also regionally referred to as Kobori Kola, Ghera Kola, Thudi Kola, and Dud Kathali Kola. Their size ranges from short to medium. A ripe version of this variety displays light yellow color on the peel. These bananas taste very sweet and often contain seeds in their body [12].	
			
	Champa Kola	Scientific name is *Musa sapientum.* Locally, it is also referred to as Chini Champa Kola. These bananas are short in size and express a golden yellow color at the Ripe stage. These bananas have a sub-acid flavor [12].	
Banana Ripeness Detection Dataset	Green	Bananas have a very firm structure at this stage. In this period, bananas convey a dark green to medium green color throughout the body. Bananas stay in this stage for 1 to 4 days. Bananas possess the highest amount of fiber and resistant starch at this level.	
	Semi-ripe	Semi-ripe bananas maintain a firm texture and depict a pale-yellow color on the peel, but the top portion of the bananas remain green. This stage lasts for 1 to 3 days. The bananas store high fiber and low carbohydrates during this period.	
			
	Ripe	At this level, bananas exhibit a bright yellow hue with brown spots on their peel. This phase lasts between 1 and 6 days. During this period, bananas reach their peak sweetness and develop a soft, easily peelable texture. Bananas contain most micronutrients such as potassium and vitamin B_6_ at this stage.	
	Overripe	Overripe bananas are fully black, or partially black, or have significant brown spots on the peel. This stage can persist for 2 to 5 days. In this phase, bananas become rich in sugar, potassium, and magnesium. They become very soft and possess a mushy interior during this period.	
			

**Fig. 3:**
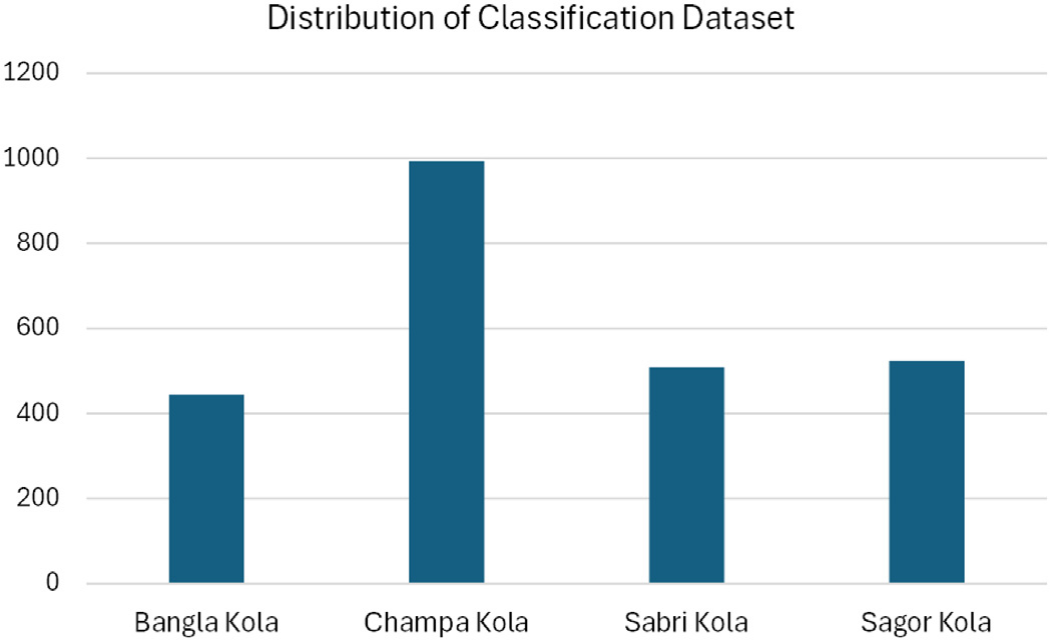
Distribution of different classes of images in the Banana Classification Dataset.

As presented in Table 2, the Banana Classification Dataset is not fully uniform. Fig. 3 shows a visual representation of the image distribution of this dataset. The class frequency counts are as follows Bangla Kola: 444, Champa Kola: 994, Sabri Kola: 509, and Sagor Kola: 524. The mean is 617.75 and the standard deviation is 219.30. The percentage representations are as follows: Bangla Kola: 18.0 %, Champa Kola: 40.2 %, Sabri Kola: 20.6 %, and Sagor Kola: 21.2 %. The proportion or ratio of the varieties is approximately as follows - Bangla Kola: Champa Kola : Sabri Kola : Sagor Kola = 1:2:1:1. This imbalance in dataset signifies that machine learning and deep learning models may become biased towards the Champa Kola variant. To address the imbalance in this dataset, data-level techniques such as oversampling the minority class, undersampling the majority class, and class weighting, and algorithm-level techniques such as class weight adjustment, cost-sensitive learning, and sampling during training can be applied.

**Fig. 4:**
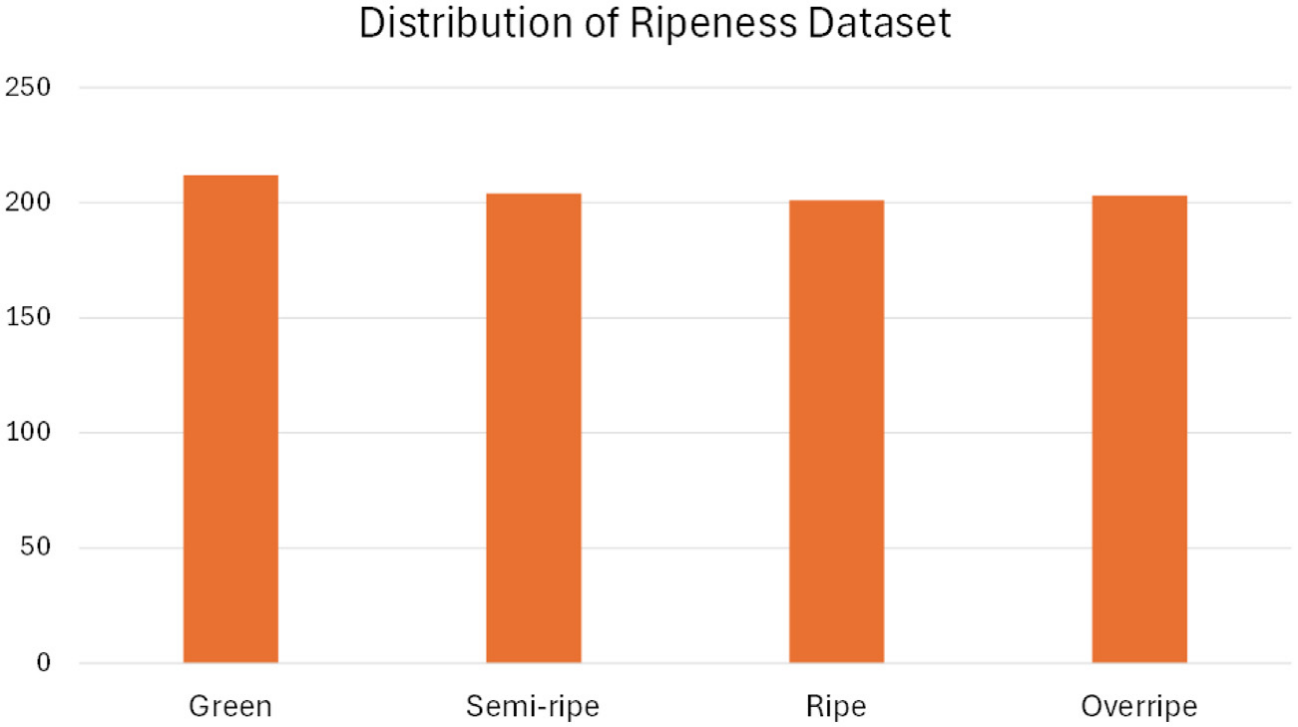
Distribution of different classes of images in the Banana Ripeness Detection Dataset.

The Banana Ripeness Detection Dataset is mostly balanced (Table 2). Fig. 4 shows a visual representation of the image distribution of this dataset. The class frequency counts are as follows Green: 212, Semi-ripe: 204, Ripe: 201, and Overripe: 203. The mean is 205.0 and the standard deviation is 4.18. The percentage representations are as follows: Green: 25.8 %, Semi-ripe: 24.9 %, Ripe: 24.5, and Overripe: 24.8. The proportion or ratio of the varieties is approximately as follows - Bangla Kola: Champa Kola : Sabri Kola : Sagor Kola = 1:1:1:1. This balanced property of this dataset ensures fair training and evaluation of machine learning and deep learning models.

## 4 Experimental Design, Materials and Methods

A step-by-step procedure has been followed to compile the dataset. Each of these steps is discussed in detail in this section.

### 4.1 Procuring bananas from markets

We visited different local markets to collect different varieties of Banana and of various maturity phases. It was made sure that the Bananas were not diseased or spoiled in any way and in their natural state. The collected bananas along with their stems were categorized considering their respective variety and ripeness.

### 4.2 Data acquisition

**Fig. 5:**
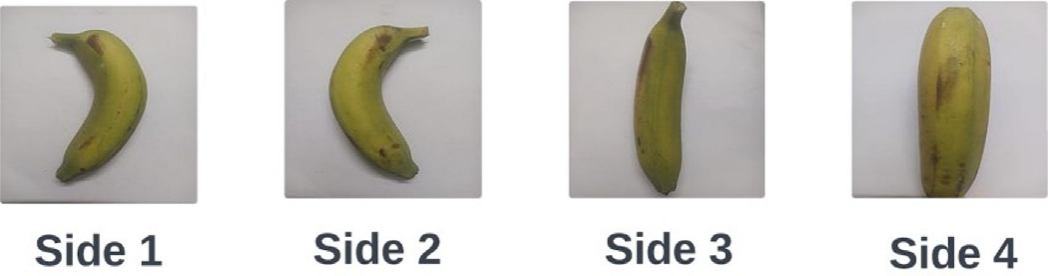
Four sides of a banana.

High-definition raw images were captured with the rear camera of an iPhone 13 Pro Max smartphone. The camera specification and image capture settings were as follows – resolution: 12 MP, aperture: f/1.5, focal length: 26 mm (wide), sensor size: 1/1.7″, pixel size on the sensor: 1.9 µm, sensitivity to light: ISO 50-100, shutter speed: 1/60 s. Bananas were detached from their stems to ensure that the entire banana is properly captured in the frame. Each banana was placed on a white paper which helped to capture the objectʼs texture, shape, and details without distortion. Furthermore, the picture was taken under a white LED light to preserve the genuine color of the bananas. It was made sure that no shadows were visible. Once the banana is correctly positioned, the image is captured from a specific height to confirm that the entire banana is visible in the frame. After photographing one side of the banana, it was rotated to capture images of the other sides of the banana. This process is repeated to take pictures of each banana from the four sides. It helps improve the datasetʼs diversity and generalization. Fig. 5 illustrates the photo capturing process of a single banana. This image acquisition procedure was rigorously followed for all bananas in the collection. Throughout the whole process, adequate lighting was maintained, and each banana was positioned precisely. To capture the different ripeness stages, bananas were photographed at four distinct stages: Green, Semi-ripe, Ripe, and Overripe. For each banana variety, images were taken daily, documenting the gradual changes in color, texture, and overall appearance as the fruit ripened. It took around 4–7 days for each banana to pass through these stages.

### 4.3 Data management

After collecting raw images, a through scrutiny was carried out on the images to identify and remove any duplicated images captured by accident. Afterwards, the raw images were uploaded on Google Drive folders and shared among the members of the data processing and augmentation team.

### 4.4 Data preprocessing

Image resizing and sharpening techniques were applied on the original images. Image resizing transforms all images into a uniform dimension, which is very significant for the batch processing technique incorporated in training Convolutional Neural Network (CNN) models. Resized images primarily offer the benefits of reduced memory usage and lower computational power requirements. Moreover, they assist machine learning and deep learning models to prevent overfitting problems by excluding redundant features from the images. An image dataset with standard dimensions, attained by reducing image resolutions, is highly convenient for storage, manipulation, and transfer. To leverage the above-mentioned advantages, all images in our dataset were resized to a standard dimension of 256×256 pixels utilizing the resize function of Python's OpenCV library.

**Fig. 6:**
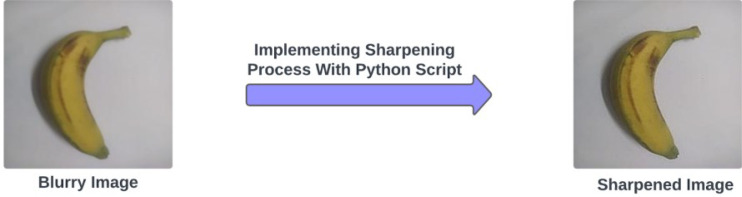
Blurry image and its corresponding sharpened image.

Eliminating blurriness from the picture elevates the entire quality of that picture and augments the feature representation of the captured object. With a Python script, an image sharpening method named Sharpness of PIL library's ImageEnhance module was applied to the blurry images with a factor of 2.0 to remove blurriness adequately. An example of the difference between a blurry image and its corresponding sharpened image is shown in Fig. 6. By completing the image preprocessing procedures, we achieved the raw version of the banana variety dataset with a combined total of 2471 images and the raw version of the ripeness detection dataset with an overall sum of 820 images.

### 4.5 Data augmentation

Data augmentation is essential for introducing data diversity and generalizing features in the datasets. In the real world, datasets cannot possibly accumulate all the variations or perspectives. Therefore, raw datasets typically contain a single version of real scenarios, leading the ML/DL models to become overfitting. Models trained on such raw image datasets of objects, donʼt acquire the ability to recognize the same objects from the images captured from different perspectives. To deal with the problem of limited data diversity, augmentation methods provide a solution by generating synthetic variations of the existing data. Using augmentation techniques, raw datasets can be enriched with variant forms of the acquired data without providing extensive labor to collect them manually from the real world. Augmented datasets expedite the computer vision models to learn the data features from multiple views, enabling the models to attain generalizability and robustness in identifying unseen data from complex real-world situations.

Several augmentation techniques have been employed combinedly on each image in our original datasets, providing the augmented datasets with two augmented images for each original image keeping including the original images as well. This results in a ratio of augmented image : original image = 2 : 1. Augmentation methods such as flipping, rotations, brightness variations, blur, and noise inclusion are applied to diversify our raw datasets. Image flipping and rotation are utilized to help models become invariant to orientation changes. Different brightness levels are introduced in our datasets to improve model robustness to various outdoor lighting conditions due to season and weather variations. Blurry and noisy versions of raw images help models generalize better and not become overly reliant on specific training data features. Implemented augmentation mechanisms are shown in Table 3.

**Table 3:** Implemented augmentation methods.

Augmentation method	Amount/Type
Flipping	Horizontal and Vertical
90° Rotation	Clockwise, Counter-clockwise, Upside-down
Rotation	Between −15° and +15°
Brightness	Between −15 % and +15 %
Blur	Up to 2.5px
Noise	Up to 1.5 % of pixels

After applying augmentation to the Banana Classification Dataset, we obtained the Augmented Banana Classification Dataset, comprising a total of 7413 images. Similarly, the Banana Ripeness Detection Dataset was augmented, resulting in the Augmented Banana Ripeness Detection Dataset with a total of 2457 images. As discussed above, a 2:1 ratio of augmentation images compared to the original images is applied in the augmentation process across all the subclasses, the quantitative metrics and statistics of the augmented datasets are same as the original dataset (please refer to Table 2 for distribution of augmented samples).

**Fig. 7:**
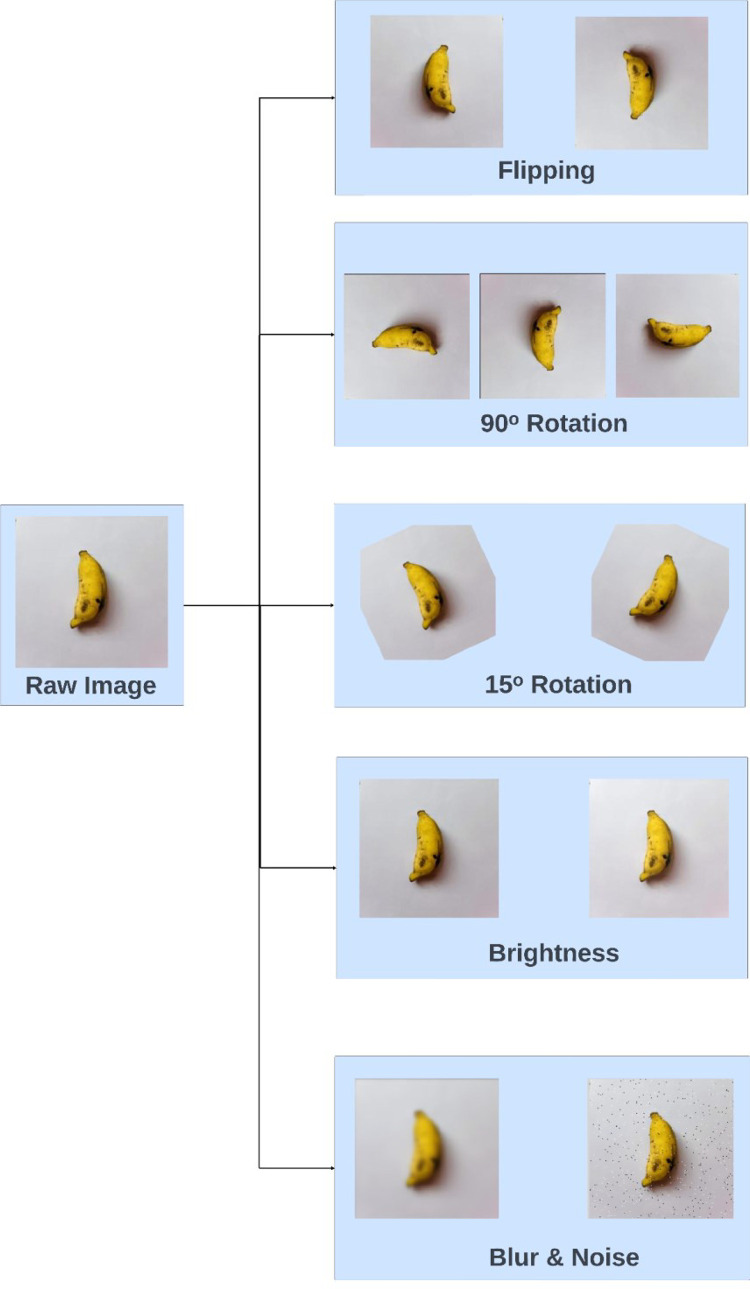
Implemented augmentation methods with sample images.

Fig. 7 shows the augmented images obtained from a sample raw image for each of the augmentation methods.

After obtaining the raw and augmented datasets, each dataset was compressed in ZIP format to reduce their storage size. Finally, we uploaded all the compressed ZIP files of our original and augmented datasets to the Mendeley Data website for public access (*Banana Classification Dataset.zip, Banana Ripeness Detection Dataset.zip, Augmented Banana Classfication.zip*, and *Augmented Banana Ripeness Detection.zip*).

## Limitations

• Weather conditions and geographical factors, such as variations in temperature, humidity, precipitation, and soil conditions, can affect the color and texture of bananas. Also, the visual features of banana skin that indicates the ripeness stages can depend upon environmental conditions and vary among varieties. Therefore, although this dataset covers most of the common banana varieties of Bangladesh, it may not be universally applicable and generalizable.
• This dataset contains images of the most common varieties of bananas cultivated and consumed throughout Bangladesh. Other banana variants, including rare and wild ones, can be found in specific geographic regions. Further data collection can be conducted to address such rare or wild varieties of bananas.

## Ethics Statement

The authors have read and followed the ethical requirements for publication in Data in Brief and confirm that the current work does not involve human subjects, animal experiments, or any data collected from social media platforms.

## CRediT Author Statement

**Md Hasanul Ferdaus:** Conceptualization**,** Formal Analysis, Validation, Writing - Review & Editing, Supervision. **Rizvee Hassan Prito:** Conceptualization, Investigation, Data Curation, Visualization, Writing – Original Draft. **Ahmed Abdal Shafi Rasel:** Resources, Investigation, Validation, Writing – Review & Editing. **Masud Ahmed:** Validation, Writing - Review & Editing. **Md. Jahid Hassan Saykot**: Data Curation, Resources. **Shanjida Sultan Shanta:** Data Curation, Resources. **Sonali Akter:** Data Curation, Resources. **Ankan Chandra Das:** Data Curation, Resources. **Mohammad Manzurul Islam:** Supervision, Project Administration. **Mahamudul Hasan:** Conceptualization, Supervision. **Md Sawkat Ali:** Resources, Supervision, Project Administration.

## Data Availability

Mendeley DataBananaImageBD: A Comprehensive Image Dataset of Common Banana Varieties with Different Ripeness Stages in Bangladesh. (Original data).

## References

[1] Chen M., Chen Z., Luo L., Tang Y., Cheng J., Wei H., Wang J. (2024). Dynamic visual servo control methods for continuous operation of a fruit harvesting robot working throughout an orchard. Comput. Electron. Agricult..

[2] Wu F., Yang Z., Mo X., Wu Z., Tang W., Duan J., Zou X. (2023). Detection and counting of banana bunches by integrating deep learning and classic image-processing algorithms. Comput. Electron. Agric..

[3] Alzate Acevedo S., Carrillo Á.J.D., Flórez-López E., Grande-Tovar C.D. (2021). Recovery of banana waste-loss from production and processing: a contribution to a circular economy. Molecules.

[4] https://www.promusa.org/Banana+diversity+in+Bangladesh. (Last Accessed: 25-Nov-2024).

[5] Sheikh M.R., Hossain M.A., Hossain M., Islam M.M., Himel G.M.S. (2024). BananaSet: a dataset of banana varieties in Bangladesh. Data Br..

[6] Piedad E.J., Caladcad J.A. (2023). Post-harvested Musa acuminata banana tiers dataset. Data Br..

[7] Arman S.E., Bhuiyan M.A.B., Abdullah H.M., Islam S., Chowdhury T.T., Hossain M.A. (2023). BananaLSD: a banana leaf images dataset for classification of banana leaf diseases using machine learning. Data Br..

[8] Mduma N., Leo J. (2023). Dataset of banana leaves and stem images for object detection, classification and segmentation: a case of Tanzania. Data Br..

[9] Medhi E., Deb N. (2022). PSFD-Musa: a dataset of banana plant, stem, fruit, leaf, and disease. Data Br..

[10] Sunitha P., Uma B., Channakeshava S., Babu S. (2023). A fully labelled image dataset of banana leaves deficient in nutrients. Data Br..

[11] M.H. Ferdaus, R.H. Prito, A.A.S.R. Rasel, M. Ahmed, M.J.H. Saykot, S.S. Shanta, S. Akter, A.C. Das, M.M. Islam, M. Hasan, S. Ali, BananaImageBD: a comprehensive image dataset of common banana varieties with different ripeness stages in Bangladesh, Mendeley Data V2. 10.17632/ptfscwtnyz.2. PMC11742353 39830620

[12] R.V. Valmayor, S.H. Jamaluddin, B. Silayoi, S. Kusumo, L.D. Danh, O.C. Pascua, R.R.C. Espino, Banana cultivar names and synonyms in Southeast Asia, Advancing Banana and Plantain R & D in Asia and the Pacific (1999). https://hdl.handle.net/10568/105378.

